# Application of Unsupervised Learning for the Evaluation of Aerogels’ Efficiency towards Dye Removal—A Principal Component Analysis (PCA) Approach

**DOI:** 10.3390/gels9040327

**Published:** 2023-04-12

**Authors:** Khaled Younes, Yahya Kharboutly, Mayssara Antar, Hamdi Chaouk, Emil Obeid, Omar Mouhtady, Mahmoud Abu-samha, Jalal Halwani, Nimer Murshid

**Affiliations:** 1 College of Engineering and Technology, American University of the Middle East, Egaila 54200, Kuwait; yahya.kharboutly@aum.edu.kw (Y.K.); mayssara.antar@aum.edu.kw (M.A.); hamdi-chaouk@aum.edu.kw (H.C.); emil.obeid@aum.edu.kw (E.O.); omar.mouhtady@aum.edu.kw (O.M.); mahmoud.abusamha@aum.edu.kw (M.A.-s.); nimer.murshid@aum.edu.kw (N.M.); 2 Water and Environment Sciences Lab, Lebanese University, Tripoli 22100, Lebanon; jhalwani@ul.edu.lb

**Keywords:** aerogels, dye removal, hydrogel, hydrogel composites, machine learning, principal component analysis, wastewater treatment

## Abstract

Water scarcity is a growing global issue, particularly in areas with limited freshwater sources, urging for sustainable water management practices to insure equitable access for all people. One way to address this problem is to implement advanced methods for treating existing contaminated water to offer more clean water. Adsorption through membranes technology is an important water treatment technique, and nanocellulose (NC)-, chitosan (CS)-, and graphene (G)- based aerogels are considered good adsorbents. To estimate the efficiency of dye removal for the mentioned aerogels, we intend to use an unsupervised machine learning approach known as “Principal Component Analysis”. PCA showed that the chitosan-based ones have the lowest regeneration efficiencies, along with a moderate number of regenerations. NC2, NC9, and G5 are preferred where there is high adsorption energy to the membrane, and high porosities could be tolerated, but this allows lower removal efficiencies of dye contaminants. NC3, NC5, NC6, and NC11 have high removal efficiencies even with low porosities and surface area. In brief, PCA presents a powerful tool to unravel the efficiency of aerogels towards dye removal. Hence, several conditions need to be considered when employing or even manufacturing the investigated aerogels.

## 1. Introduction

Climate change is believed to make the freshwater scarcity problem more dramatic, both in the water-stressed regions and at the globe’s level. When greenhouse gases (GHG) accumulate, the atmosphere’s temperature increases, causing a definite hamper to the water cycle. This anthropogenic act of warmer atmospheres will definitely present an input on the accelerated melting of sea ice and glaciers. This returns water that has been locked up for thousands of years effect back into the dynamic water cycle. On the other hand, higher temperatures will increase the rate of vaporization for the newly introduced water. Because water in the gas state is considered a GHG, increased evaporation leads to increased warming. This phenomenon is known as the positive feedback loop. Additionally, increased amounts of atmospheric water vapor and heat energy can combine to cause events such as hurricanes. It may seem counterintuitive, but big storms can actually intensify water scarcity. When too much water arrives all at once, much of it will simply run off, leaving aquifers unreplenished. On top of that, a big hurricane can also cause extensive infrastructure damage and contamination that further worsens water scarcity [1].

As can be seen above, different natural and/or anthropogenic effects could lead to a lack of fulfillment in the drinking water needs for the international population. Overcoming this issue requires the development and engagement of more sophisticated and optimized water treatment techniques. These techniques will allow us to increase water accessibility, therefore targeting SDG6 (Clean Water and Sanitation), in a direct way. On the other hand, attempting this also targets other SDGs in an indirect way, such as SDG2 (Zero Hunger), SDG3 (Good Health and Well-Being), SDG11 (Sustainable Cities and Communities), SDG13 (Climate Change), SDG 14 (Life Below Water), and SDG15 (Life on Land) [2].

Multiple water treatment techniques exist, based on different physical, chemical, and even biological principles. Physical guided approaches are commonly referred to as the “pretreatment” steps of water purification. They simply involve bar screens, which are simply large vertical bars that stand at the inlet of nearly every waste treatment plant, designed to stop larger objects from getting into the plant and hurting machinery such as pumps. Grit chambers are the next step in the pretreatment process, following bar screens. Since the latter cannot catch everything, larger particles called grift still need to be removed as they make the water effluent more homogenous. The velocity of rather viscous pollutants is adjusted to allow for particles such as sand and rock to settle out. This is needed because these particles cannot be removed using chemicals, and they could potentially clog or destroy pumps and membranes later on in the process. Following the grit chamber, water moves on to the primary treatment process, which starts with a large basin called the primary clarifier. It functions on the principle of settling velocity; this term can be simply defined as the speed at which a particle settles. At this step of the process, the slightly treated waste, which is referred to as effluent, is free of solids larger than 10 µm and should be all organic matter, which will be treated further [3,4]. The top layer of the clarified water flows over a weir wall and into the next basement process called the aeration basin. At this point begins the secondary treatment processes. The aeration basin’s sole focus is to significantly degrade the biological content of the sewage. The effluent flows into the basin at the bottom of which are hundreds, if not thousands, of tiny air blowers that create bubbles through the water. The water is pumped into the tank, along with activated sludge. This introduction of significant amounts of bacteria along with the massive amounts of oxygen injected from the bubblers creates an environment perfect for the process of aerobic digestion. Following aeration basins, the effluent along with the activated sludge is pumped into a secondary filter or clarifier. Water at the downstream of secondary processes moves on to the disinfection process. This is usually accomplished in one of three ways: chlorine, ozone or UV light disinfection. At this point, nearly 85% of all organic matter is removed from the water and, to an extent, could rise, depending on the need of the treatment. The water will either be discharged in nature, or it will undergo tertiary treatment so that it can be employed for drinking and domestic uses. Tertiary treatment compiles the sets of membrane technology applied for treating water. Several approaches can rise, following the particle dissolved in water size: UltraFiltration (UF), NanoFiltration (NF), and Reverse Osmosis (RO). The latter removes up to 99% of the water contaminants [3,4].

Adsorption and size exclusion present the primary concepts involved in water decontamination via membranes technology [5,6,7]. Several physical properties are questioned in order to estimate the efficiency of a certain membrane, such as porosity, surface area, and volumetric capacity of the membrane. Aerogels are gaining high relevance in water and air purification, as they are considered good adsorbents, following their high porosity (~99% [7,8]) and their high specific area (100~1600 m^2^/g [8]). In addition, these nanomaterials are characterized as lightweight, given their density that is close to the one of air (10–260 kg/m^3^; [7,8,9]). At the commercial level, silica type aerogels have been developed and widely used [10,11]. Several shortcomings may rise from their application, as they are considered rigid and fragile [12]. On the other hand, a high risk of secondary pollution (organic and inorganic) may occur from using such aerogels materials. Components such as Metal-Organic Frameworks (MOFs), Polyaniline (PA), and Poly-vinylidene-fluoride (PVDF) ([13,14]) are some of the aerogel materials that are currently employed and present as non-biodegradable nature. Therefore, their occurrence in water as secondary pollutants is problematic and tedious to handle [15]. In order to overcome such problems, the inclusion of “green” aerogels such as nanocellulose (NC) and chitosan (CS) is gaining high attention. Besides their eco-friendly character, these materials-based aerogels possess the required mechanical strength and flexibility to make them good candidates for substituting silica and other non-biodegradable polymers-based membranes [6,16]. A pile of applications can be mentioned for these materials along with graphene (G) oxide aerogels. Their scope of applications is wide due to their peculiar physical properties such as high porosity, low density, high surface area, and even biodegradability [6,17,18].

G-based and NC-based aerogels are lightweight and highly porous materials with numerous potential applications. Their high surface area and excellent electrical conductivity make them a promising tool for energy storage, such as in supercapacitors and batteries and thermal insulation in buildings [19,20,21]. These aerogels, along with the CS-based ones, exhibit low thermal conductivity and high thermal stability, making them suitable for use as insulation materials in building and industrial equipment [22,23,24]. In addition, their unique properties make these materials promising for use in biomedical applications such as drug delivery, tissue engineering, and biosensors [25,26,27]. Along their large surface area, the high catalytic activity of these aerogels makes them ideal catalysts for several petroleum and chemical industries [28,29]. For water treatment systems, their porous structure makes them ideal for water purification applications, as they can be used as adsorbents for heavy metals, organic pollutants, and bacteria [6,30]. Nanocellulose fibers and carbonized wood-based aerogels and their efficiency in dye removal have been investigated by several researchers [31,32]. 

In this study, we will focus on the applicability extent of these three families of aerogels in water treatments systems. In order to do so, we apply an unsupervised machine learning technique, the so-called “Principal Component Analysis” (PCA), for the sake of estimating the efficiency of dye removal along the three aforementioned types of aerogels (NC-, CS-, and G-based aerogels). As far as our knowledge goes, this study presents the first attempt in applying PCA methodology for the sake of estimating these aerogels’ efficiency, towards dye removal, in a comprehensive way.

## 2. Results and Discussion

PCA analysis was conducted and plotted based on previously published data (Table 1) from the study of Paul and Ahankari [6].

**Figure 1:**
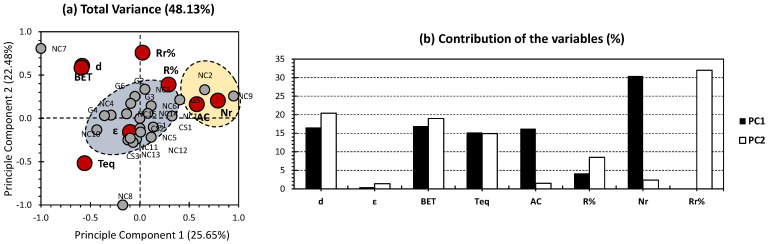
PCA-biplot for all datasets. Grey bullets represent the individuals of the population (different investigated nanocellulose-based aerogels). Red bullets represent the variables (different employed physical/chemical and adsorption parameters). The yellow cluster exclusively gathered NC1 and NC9 while the blue cluster encompasses the rest of the nanocellulose-based aerogels.

Figure 1 shows the PCA results for the previously published findings on the physical/chemical and adsorption parameters of several nanocellulose (NC)-, chitosan (CS)-, and Graphene (G)-based aerogels involved in water purification [6]. The first two PCs showed 48.13% of the total variance (25.65% for PC1 and 22.48% for PC2; Figure 1a). Even though an average variance has been shown, it could be employed to decipher any correlation between the groups of the investigated parameters. The yielded proportion of variance could occur from either a low correlation between the investigated variables (chemical and physical parameters, from one side, and adsorption parameters, from another side) or the occurrence of additional physical and chemical features that influence the adsorption behavior of the investigated nanocellulose-based aerogels. The first is more likely discarded since the chemical component and physical textures compose the features that influence interactivity and reactivity of any component, in a synergetic way. The second hypothesis is more likely to be closer to the truth, as several properties (such as permeability, tortuosity, molecular diffusivity, etc.) are not being included in the investigations. For the variables, the number of reuse/regeneration (referred to as Nr in all figures) presented the highest contribution along PC1, accounting for 30.46% (Figure 1b). Density, BET surface area, time to reach equilibrium, and adsorption capacity (referred to as AC in all figures) presented a moderate contribution along PC1, accounting for 16.54%, 16.93%, 15.17%, and 16.48%, respectively (Figure 1b). As for PC2, removal efficiency after regeneration (Rr%) exhibited the highest contribution, scoring for 32% (Figure 1b). Similar to the trends of PC1, density, BET surface area, and time to reach equilibrium (referred to as Teq in all figures) exhibited moderate contribution, scoring for 20.40%, 18.97%, and 14.89%, respectively. The high contribution of these factors could be reliable since no lack of their data has occurred, in a frequent way. Density is the only moderately weighted parameter that could be exempted from the aforementioned statement, following the lack of data in-hand (Table 1). Following the yielded trends, it can be said that both PCs were simultaneously influenced by most of the investigated physical parameters (all except for porosity). For the adsorption parameters, PC1 most likely presents number of reuse/regeneration, and PC2 most likely presents removal efficiency after regeneration (Rr%). Interestingly, both of the aforementioned adsorption parameters were almost equally influenced by the same physical properties.

**Figure 2:**
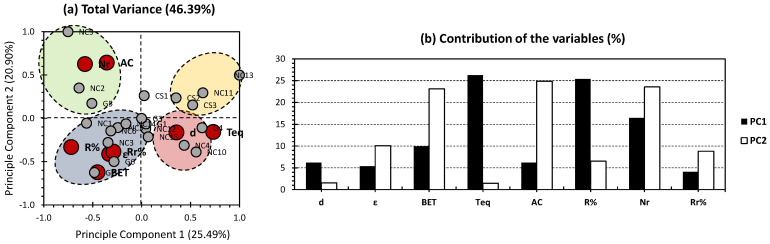
PCA-biplot for all datasets, with the exclusion of NC7 and NC8. Grey bullets represent the individuals of the population (different investigated nanocellulose-based aerogels). Red bullets represent the variables (different employed physical/chemical and adsorption parameters). The yellow cluster gathered the chitosan-based aerogels (CS1, CS2, and CS3); the blue cluster gathered nanocellulose-based aerogels NC1, NC3, NC5, NC6, and NC14 and graphene-based aerogels G2, G3, and G6; the green cluster gathered aerogels NC2, NC9, and G5; the red cluster gathered nanocellulose-based aerogels NC4, NC10, NC12, and NC15 and graphene-based aerogels G1, and G4.

For the individuals, two different clusters can be distinguished (blue and yellow; Figure 1a). The yellow cluster exclusively gathered NC2 and NC9 and showed a high positive correlation along adsorption capacity and number of reuse/regeneration, which are both part of the investigated adsorption parameters panel (Table 1). Interestingly, NC2 and NC9 showed no or a negative influence according to the physical parameters. In fact, no samples showed tremendous influence along porosity, as it is yielded near the origin (Figure 1a). The other physical parameters were plotted on the negative side of PC1, showing a negative influence on the two investigated nanocellulose-based aerogels. These findings make sense, as the yielded data present average to low values, in comparison with their homologues (Table 1). This probably indicates that NC2 and NC9 could only be employed in cases such as those with a long time to reach equilibrium, a low BET surface area, and where low density can be handled. The blue cluster encompasses the rest of the nanocellulose-based aerogels, with the exception of NC7 and NC8. For the aforementioned nanocellulose-based aerogels, they both showed a negative trend, along PC1, with relatively high positive and negative influences along PC2 for NC7 and NC8, respectively. The components of the blue cluster are most likely located around the node; this indicates their lack of contribution in the PCA factor loadings. Most of the loading was actually for NC7 and NC8. This magnificent difference in the factor loadings’ magnitude skewed the clusters’ arrangement, when the whole dataset has been taken into account (Figure 1). In order to overcome this issue, we have attempted to run a PCA investigation without NC7 and NC8 (Figure 2).

Figure 2 shows the PCA results of the investigated aerogels, with the exception of NC7 and NC8. The first two PCs showed 46.39% of the total variance (25.49% for PC1 and 20.90% for PC2; Figure 2a). Even though a slightly lower representativeness was obtained in this case, if compared to the all-dataset approach (Figure 1a), fewer aerogel samples were projected near the node of the first two PCs (Figure 2a). In addition, a relatively higher dispatchment was obtained between the investigated aerogels, as four clusters were identified. These findings prove the effectiveness of the adopted approach, as it removed the bias yielded from the overly loaded factors in the case of the inclusion of NC7 and NC8. For the variables, the time to reach equilibrium and Removal Efficiency (R%) presented the highest contributions along PC1, accounting for 26.25%, and 25.42%, respectively (Figure 2b). Number of reuse/regeneration exhibited a relatively lower contribution, accounting for 16.46% (Figure 2b). This indicates the high influence of time to reach equilibrium (as a “Physical property”) on the Removal Efficiency (R%) (as an “adsorption property”). As for PC2, the highest contributions were yielded for BET surface area, adsorption capacity, and Number of reuse/regeneration (23.13%, 24.86%, and 23.58%, respectively; Figure 2b). This indicates the high influence of BET surface area (as a “Physical property”) on the adsorption capacity, and Number of reuse/regeneration (Nr) (as an “adsorption property”). In order to confirm a negative or positive influence, a closer look-up on the PCA bi-plot should be taken into consideration (Figure 2a). Following the trends of the different variables along the first two PCs, time to reach equilibrium and removal efficiency (R%) were plotted on opposite sides, as the first is found to be on the negative part of PC1, and the second is found on its positive part. These findings make sense, as the lower the time to reach equilibrium is, the more thermodynamically favorable the interaction between the aerogel and the pollutant to be treated will be; therefore, the more favorable the side of the reaction towards removing the pollutant will be.

For the individuals, four different clusters can be distinguished: upper right (yellow), upper left (green), lower left (blue), and lower right (red) (Figure 2). The yellow cluster gathered the chitosan-based aerogels (CS1, CS2, and CS3) along with two nanocellulose-based aerogels (NC11 and NC13). This cluster was localized on the positive sides of both PCs. Its components showed no accordance along any of the physical and adsorption parameters. This probably indicates that, between the whole investigated aerogels, the components of this cluster showed the lowest efficiency. Interestingly, chitosan aerogels showed the lowest regeneration efficiencies, along with a moderate number of regeneration (Table 1). The green cluster gathered aerogels NC2, NC9, and G5 and was localized on the negative side of PC1 and the positive side of PC2 (Figure 2a). The components of this cluster showed a high accordance with adsorption capacity and number of reuse/regeneration; this indicates that these aerogels are most likely influenced by porosity and BET surface area as physical parameters (check Figure 2b for the high influence of the two last parameters on PC2). The aforementioned statements could give an indication that NC2, NC9, and G5 are to be employed in cases for dyes with a high adsorption energy to the membrane and where a high porosity could be envisaged. In addition, the scope of application of these aerogels should tolerate lower removal efficiencies. The blue cluster gathered nanocellulose-based aerogels NC1, NC3, NC5, NC6, and NC14 and graphene-based aerogels G2, G3, and G6 and was localized on the negative sides of both PCs (Figure 2a). The components of this cluster showed a high accordance along the removal efficiency before (R%) and after regeneration (Rr%) adsorption parameters; for the physical parameters, and oppositely to the green cluster, these aerogels are most likely influenced by low porosity and BET surface area (check Figure 2b for the high influence of the two last parameters on PC2). The aforementioned statements could give an indication that the following aerogels could present high removal efficiencies even with low porosities and surface area; therefore, considering a manufacturing process with low functionalization rate of the membrane could be acceptable. The red cluster gathered nanocellulose-based aerogels NC4, NC10, NC12, and NC15 and graphene-based aerogels G1, and G4 and was localized on the positive side of PC1 and the negative side of PC2 (Figure 2a). The components of this cluster showed a high accordance along with density, and time to reach equilibrium. The lack of accordance with adsorption parameters, especially those related to the removal efficiency and the number of reuse/regeneration, probably indicates that the components of this cluster are not as suited for dye contamination, as those of other clusters.

Several types of aerogels exist and exhibit quite an adsorption capacity towards dye and other contaminants’ removal. For detailed discussion and more examples, readers can refer to Paul and Ahankari’s review paper [6]. In this study, our investigations focused on the different samples indicated and collected in Table 1. In fact, adding even one dataset could be responsible for hampering the whole trend of the correlations. The aforementioned statement could be proven by the difference of trends yielded between Figure 1 and Figure 2. Following these statements, two shortcomings could rise from the data-driven model applications. The first is the potentially high bias that could occur in case of addition of only one dataset. The second is the limitation in the number of individuals that could be employed, for the sake of reducing the bias in the dataset and keeping the variance as high as possible.

## 3. Conclusions

In this study, we applied an unsupervised machine learning technique, the “Principal Component Analysis” (PCA), for estimating the efficiency of dye removal along several types of aerogels. The three families of investigated aerogels are the nanocellulose (NC)-based, chitosan (CS)-based, and graphene (G)-based. A full data analysis shows that most of the dataset components were clustered around the node of the PCA-biplot of the first two Principal Components (PCs). In order to overcome this obstacle, the two samples (NC7 and NC8) that encompassed most of the factor loadings were discarded. Attempting this strategy allowed to “stretch out” the investigated dataset, as samples were more dispatched around the node of the PCs, in one side, and between each other, in another side. This shows that the efficiency and scope of applicability of PCA or any data analysis technique strongly depends on the organization of the investigated dataset, and is not only restricted to the shortcomings of the method itself. In brief, a good data pre-treatment is indispensable when considering data mining techniques.

For the different investigated samples, the yielded trends have shown that, even though some similarities at the molecular level exist between several aerogels (check Table 1), it does not strictly mean that their behavior at the technical scale will be similar. Several discrepancies may occur, following the reliance of the removal efficiency of dyes contamination by aerogels on the physical and adsorption parameters. Hence, the microscopic scale along with the mesoscopic one will both have their influence on the macroscopic behavior and, therefore, the technical performance. Furthermore, PCA revealed the high influence of the time to reach equilibrium (as a “physical property”) on the removal efficiency (R%) (as an “adsorption property”). This logical finding ascertains the applicability of PCA to compare between different types of aerogels. In addition, PCA unraveled a high influence of BET surface area (as a “physical property”) on the adsorption capacity, and the number of reuse/regeneration (as an “adsorption property”).

For the comparison between the three investigated groups of aerogels, the chitosan-based ones showed the lowest regeneration efficiencies, along with a moderate number of regenerations. NC2, NC9, and G5 aerogels are more likely to be used where there is high adsorption energy to the membrane, and high porosities could be tolerated. In addition, the scope of application of these aerogels should allow lower removal efficiencies of dye contaminants. NC3, NC5, NC6, and NC11 could present high removal efficiencies even with low porosities and surface area. Hence, considering a manufacturing process with low functionalization rate of membranes containing such nanocellulose-based aerogels could be applicable.

In brief, the data-driven approach presents a powerful tool to seek the efficiency of aerogels towards dye removal, as several conditions are to be considered when employing or even manufacturing these aerogels. In fact, the authors are currently working on another project on evaluating the efficiency of aerogels for removal of ions from wastewater [56]. Even though this method appears as an efficient one to trace similarities and dissimilarities, one should be cautious when using it, as it hides some parts of the whole image, since the total variance is rarely at its maximum.

## 4. Methodology

Principal Component Analysis (PCA) is a statistical technique used to reduce the complexity of a dataset by transforming it into a smaller set of uncorrelated variables called principal components (PCs). PCA is commonly used in data analysis and machine learning to extract meaningful information from large datasets with many variables [57]. The main idea behind PCA is to find a new set of variables that are linear combinations of the original variables, and that capture as much as variables in the dataset as possible [57]. The first PC is the direction in the data that has the highest variance, and each subsequent PC is chosen to be orthogonal to the previous components and to have the highest possible variance subject to that constraint [57]. This method has found its applicability in simplifying the analysis of large datasets by reducing the number of variables while retaining the most important information. It can also help identify patterns and relationships in the data, as well as outliers and anomalies [58]. Nonetheless, PCA has few limitations, as it assumes that the data are linearly related and may not perform well if non-linear relationships or complex structures exist. In addition, PCA can be sensitive to outliers which can give biased results to the analysis [58].

### 4.1. Data Collection and Pre-Treatment

Data have been collected from the published study of Paul and Ahankari [6]. Table 1 presents the inventory of the different investigated NC-, CS-, and G-based aerogels, along with their performance capacity, adsorption parameters, and physical/chemical characteristics.

The data of each of the investigated variables has a different weight. In order to remove any bias yielded by the difference of magnitude, a normalization technique similar to the one of Younes et al. [59] has been adopted as follows:

$$Y_{st}=\frac{\left(Value-Mean\right)}{Standard\;Deviation}$$

where “*Y_st_*” presents the standardized dataset values.

### 4.2. Principal Component Analysis (PCA)

We have used similar methodology that we used in our previously published work [60,61,62]. After normalization, PCA findings were yielded by using XLSTAT 2014 software, following the similar approach adopted by Younes et al. [60,61,62]. In this study, the missing data were estimated using a built-in feature that replaces a missing value with the “Mode”, following the respective variables.

The aim of this study is to apply PCA on the data found in a previous study by Paul and Ahankari [6] (Table 1). Applying PCA targets at searching for any hidden layers between the physical/chemical properties from one side and adsorption parameters from another side. In case it occurs, this will help in the better interpretation and, therefore, understanding of different factors that influence the applicability of a certain aerogel membrane. The output information yielded by PCA could help in several stages of the water treatment process, from the manufacturing approach to the experimental conditions and to the removal efficiency of a selected membrane. Here, we have applied PCA for 8 different factors, influencing 24 investigated aerogels (Table 1). PCA is a data-driven unsupervised machine learning technique that works on the reduction of a certain dataset. The outcome of such reduction has been applied for a better visualization of a certain phenomenon, the seeking of a hidden knowledge by the given correlations (negative or positive), and the representativity of the Principal Components (PCs) to the population in-hand. The j^th^ PC matrix (*Fi*) is expressed using a unit-weighting vector (*U_j_*) and the original data matrix M with m x n dimensions (m: number variables, n: number of datasets) as follows [63,64,65,66]:

$$Fi=U_{j}^{T}M=\sum_{}^{i=0}U_{ji}M_{i}$$

where *U* is the loading coefficient, and *M* is the data vector of size n. The variance matrix *M*(*Var*(*M*)) is obtained by projecting *M* to *U* and should be maximized as follows:

$$Var\left(M\right)=\frac{1}{n}\left(UM\right){\left(UM\right)}^{T}=\frac{1}{n}{UMM}^{T}U$$

$$MaxVar\left(M\right)=Max\left(\left(\frac{1}{n}\right){UMM}^{T}U\right)$$

Since $\frac{1}{n}{MM}^{T}$ is the same as the covariance matrix of *M*(*cov*(*M*)), *Var*(*M*) can be expressed as follows:

$$Var\left(M\right)=U^{T}cov\left(M\right)U$$

The Lagrangian function can be defined by performing the Lagrange multiplier method as follows:

$$L=U^{T}$$

$$L=U^{T}cov\left(M\right)U-\delta (U^{T}U-1)$$

“*U^T^U* − 1” is considered to be equal to zero, since the weighting vector is a unit vector. Hence, the maximum value of *var*(*M*) can be calculated by equating the derivative of the Lagrangian function (*L*) in respect to *U* as follows:

$$\frac{dL}{dU}=0$$

$$cov\left(M\right)U-\delta U=\left(cov\left(M\right)-\delta I\right)U=0$$

where *δ* is the eigenvalue of *cov*(*M*) and *U* is the eigenvector of *cov*(*M*).

## Figures and Tables

**Table 1:** Nanocellulose (NC)-, chitosan (CS)-, and Graphene (G) oxide-based aerogels in dye removal; physical/chemical and adsorption parameters. (Adapted from Paul and Ahankari Ref. [6], copyright (2023), with permission from Elsevier).

Sl. No.	Aerogel Composition	Physical/Chemical Parameters				Adsorption Parameters				Ref.
		Density (mg/cm^3^)	Porosity (%)	BET surface area (m^2^/g)	Time to reach equilibrium (min)	Adsorption capacity (mg/g)	Removal Efficiency (%)	Number of reuse/regenerations	Removal efficiency (%) after Regeneration	
Nanocellulose (NC)-based Aerogels										
1	SPCNF	2.1	–	–	5	222.2	99	–	–	[33]
2	CNF/PEI/ Ag NPs	14.9	96.5	3.5	5	–	99.2	10	98	[34]
3	GO/CNF	26.4	98.2	–	20	–	99	5	91	[16]
4	GO/CNF	26.3	–	35	360	111.2	–	3	98	[35]
5	GO/CNF	2.2	99	–	–	265.6	–	–	–	[36]
6	BNC/MoS_2_	–	–	137	120	–	96	6	86	[37]
7	UiO-66/NC	51	–	826	360	71.7	–	4	92	[38]
8	BHA	8.2	99.4	54	720	531	–	5	2	[39]
9	NB/DANC/CMC	–	–	48.6	40	29,842	–	10		[40]
10	PDA/CNF/PEI	25	98.5	–	720	265.9	–	4	89	[41]
11	Cu_2_O/TiO_2_/CNF/rGH	–	–	16.2	120	–	85.62	4	79.5	[42]
12	TEMPO-oxidized NC/CS	8.4	99	–	360	136.6	91.8	–	–	[43]
13	CMC/CNFs	14.4	93	9.8	720	917.4	>90	6	–	[44]
14	MOF-199@ CNCA/CMCS	–	–	102.6	60	1112.2	–	–	–	[45]
15	CNC-PEI-CD/PAM	–	–	–	90	155.9	95	3	85	[46]
Chitosan (CS)-based Aerogels										
1	Fe_3_O_4_@ PDA/CMC	–	–	106.7	120	217.4	93.9	7	60	[47]
2	ZnBDC/CSC	–	–	16.3	25	202	~88	5	84	[48]
3	Fc-CS	–	–	5	480	1141	92.8	5	80	[49]
Graphene (G)-based Aerogels										
1	PFGA	7.1	–	–	270	3059.2	–	4	–	[50]
2	PVA/N/GA	–	–	210.4	240	217	98.4	5	97.7	[51]
3	TCGA	–	–	–	–	-	–	–	–	[52]
4	LEGA	33.9	–	–	402	332.2	90.3	5	85	[53]
5	GA	–	–	109	15	76	–	10	89.5	[54]
6	Fe_3_O_4_/rGO	–	–	200.4	180	163.8	–	5	98	[55]

## Data Availability

All data are included in the manuscript.
